# Cyclodepsipeptides from Marine Sponges: Natural Agents for Drug Research

**DOI:** 10.3390/md8030810

**Published:** 2010-03-22

**Authors:** Gowri Shankar Bagavananthem Andavan, Rosa Lemmens-Gruber

**Affiliations:** Department of Pharmacology and Toxicology, University of Vienna, Althanstrasse 14, A-1090 Vienna, Austria; E-Mail: gowri.bagavananthem.andavan@univie.ac.at

**Keywords:** cyclodepsipeptides, papuamides, jasplakinolide, actin polymerisation, HIV entry inhibitors

## Abstract

A number of natural products from marine sponges, such as cyclodepsipeptides, have been identified. The structural characteristics of this family of cyclic peptides include various unusual amino acid residues and unique *N*-terminal polyketide-derived moieties. Papuamides are representatives of a class of marine sponge derived cyclic depsipeptides, including callipeltin A, celebesides A and B, homophymine A, mirabamides, microspinosamide, neamphamide A and theopapuamides. They are thought to have cytoprotective activity against HIV-1 *in vitro* by inhibiting viral entry. Jasplakinolide, a representative member of marine sponge-derived cyclodepsipeptides that include arenastatin A, geodiamolides, homophymines, spongidepsin and theopapuamides, is a potent inducer of actin polymerization *in vitro.* Although actin dynamics is essential for tumor metasasis, no actin targeting drugs have been used in clinical trials due to their severe cytotoxicity. Nonetheless, the actin cytoskeleton remains a potential target for anti-cancer drug development. These features imply the use of cyclodepsipeptides as molecular models in drug research.

## 1. Introduction

Cyclodepsipeptidic secondary metabolites produced by marine and terrestrial organisms have unique structures comprised of unusual amino acids and non-amino acid moieties. The novel structural features and the wide spectrum of biological activities of these peptidic metabolites have generated considerable interest. However, it is rather difficult to isolate sufficient quantities of these metabolites for pharmacological and toxicological testing. Therefore, structural determination and total syntheses of these natural compounds are required. Progress towards the synthesis and study of potential drugs of different chemical structures, isolated from various marine organisms, has been extensively reviewed [1–22]. This review will focus on cyclodepsipeptides derived from marine sponges. A series of potent HIV inhibitory cyclic depsipeptides have been described from a number of marine sponges, as well as numerous compounds, which exhibit potent cytotoxic properties *in vitro*, and this will be discussed in this review.

The HIV replication cycle offers multiple targets for chemotherapeutic intervention, including the viral exterior envelope glycoprotein gp120, viral co-receptors CXCR4 and CCR5, transmembrane glycoprotein gp41, integrase, reverse transcriptase and protease. Most currently approved anti-HIV drugs belong to nucleoside/nucleotide reverse transcriptase inhibitors, non-nucleoside reverse transcriptase inhibitors or protease inhibitors. Highly active antiretroviral therapy, which combines several drugs, has dramatically improved patient lives. However, adverse side effects, long-term toxicity and drug resistance limit their therapeutic effect. In addition to the above-mentioned drugs, compounds that target viral entry and virus-cell fusion have great potential for the treatment of HIV infections. The sponge-derived cylodepsipeptides papuamide and mirabamides have been shown to inhibit HIV entry into cells [23,24]. HIV entry into host cells is a multi-step process that remains to be fully elucidated. The proteins involved in the entry process have become attractive targets for drug design. Natural probes such as depsipeptides, which target viral entry, could provide valuable information for computer-aided drug design. In this context, recent achievements in the treatment of HIV infection and the application of computational methods for current drug design was recently reviewed [25].

Most currently used anticancer drugs, in particular those that are cytotoxic, affect the defining characteristic of cancer cells, namely the process of cell division. Thus, these drugs are antiproliferative, but they have no specific inhibitory effect on invasiveness, the loss of differentiation or the tendency to metastasize. However, a number of recently developed drugs, such as receptor tyrosine kinase inhibitors and monoclonal antibodies, affect specific targets associated with tumor cells or endothelial cells. Cyclodepsipeptides are currently under evaluation in clinical trials in patients with cancer refractory to standard therapy. Kahalalide F and aplidin (plitidepsin) have shown promising results in phase I and II clinical trials, especially when administered in combination with other cytotoxic agents. Kahalalide F was isolated from the mollusks *Elysia rufescens* and *Spisula polynyma*, and from the green alga *Bryopsis* sp. [26]. In phase I clinical trials, kahalalide F provided a benefit to patients with advanced androgen-refractory prostate cancer and other advanced tumors [27,28]. Aplidin (plitidepsin) is a cyclodepsipeptide originally isolated from the Mediterranean tunicate *Aplidium albicans*, and has been demonstrated to have strong anticancer activity against a variety of cultured human cancer cells and in tumor-bearing nude mice. Phase I and II clinical trials of aplidin have yielded promising results in cancer patients [29–38]. These examples show that cyclodepsipeptides may be of interest in the development of new agents and combination therapies with different mechanisms of action.

## 2. Compounds with Anti-HIV Activity

### 2.1. Structural characteristics

**Figure 1:**
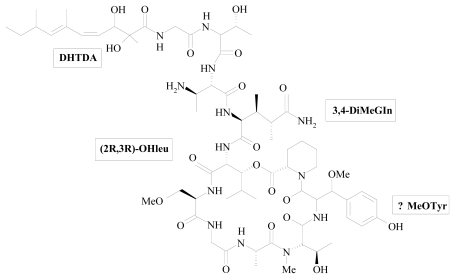
Structure of papuamide A.

The antiviral cyclic depsipeptides neamphamide A, callipeltins, papuamides and mirabamides have been isolated from various marine sponges. Papuamide A’s activity is representative of the group of marine depsipeptides shown to inhibit HIV induced cytopathicity. Papuamide A (Figure 1) shares many chemical features with this group including an aliphatic tail, depsipeptide cyclisation, a 3,4-dimethylglutamine residue and an available tyrosine hydroxyl group, which is glycosylated in mirabamides. The distinguishing structural characteristics of this family of cyclic peptides include a preponderance of unusual amino acid residues and unique *N*-terminal polyketide-derived moieties (Table 1). For instance, the atypical amino acid residues 3,4-dimethyl-l-glutamine and β-methoxytyrosine are common to all of the above mentioned sponge-derived cyclodepsipeptides, but to date have not been described elsewhere. As a result, syntheses of structural subunits such as 3,4-dimethyl-l-glutamine [39], 4-amido-7-guanidino-2,3-dihydroxyheptanoic acid [40] and 3-hydroxy-2,4,6-trimethylheptanoic acid [41] have provided the essential building blocks for use in total synthesis of the peptides, and have also helped to define the absolute stereochemistry of several key chiral centers. However, synthetic efforts to prepare intact natural products have been hampered by unresolved questions concerning the stereochemistry of β-methoxytyrosine residues, which occur throughout this family of peptides. While stereoselective syntheses of all four diastereomers of β-methoxytyrosine have been reported [42,43], the absolute stereochemistry of this residue in natural peptides was not established. Decomposition of β-methoxytyrosine during acid hydrolysis of the parent peptide has prevented the successful application of standard techniques such as Marfey’s analysis. Oku *et al*. [44] described the complete stereochemical assignment of neamphamide A and a general method for determining the absolute stereochemistry of β-methoxytyrosine residues in other peptides. This technique was also used to define the stereochemistry of the same residue in papuamide B, which was determined as (*R*)-β-methoxy-d-tyrosine. So far, such efforts have led to the successful establishment of the stereochemistry of some stereogenic centers within this class of cyclodepsipeptides [1,5,45–47]. However, none of these molecules had been assembled until Xie *et al.* [48] reported their synthetic strategy towards papuamide B. This features stereoselective assembly of its dienoic acid fragment and an efficient elaboration of the (2*S*,3*S*,4*R*)-3,4-dimethylglutamine residue through a 3,4,5-trisubstituted δ-valerolactone. This latter approach may benefit total synthesis of other anti-HIV cyclic depsipeptides such as callipeltin A and neamphamide A. The development of synthetic strategies will support large-scale syntheses of related natural products and their analogs, which would prompt structure-activity relationship studies.

### 2.2. Mode of action

Because viruses share many metabolic processes with the host cell, it is difficult to find drugs that are selective for the viral pathogen. However, there are some enzymes that are virus specific and these are potential drug targets. Most currently available antiviral agents are only effective during viral replication. The HIV virus uses the CD4 co-receptor and the chemokine receptor CCR5 or CXCR4 as binding sites.

Transcription occurs when the T cell is activated. Transcription of both the host cell DNA and the provirus is initiated by the transcription factor NF-κB. The action of the viral protease involves the cleavage of polypeptides into structural proteins and enzymes for the new virion. Currently, available drugs inhibit nucleotide or non-nucleotide reverse transcriptase or protease. Reverse transcriptase makes double-stranded DNA copies of viral RNA, and protease acts on immature virions after budding. The combination of these two classes of anti-HIV drugs has improved dramatically the prognosis of HIV/AIDS patients. However, the regimen is complex and has many unwanted effects. For example, the virus is not eradicated, but rather remains latent in memory T cells, and can become reactivated if the drugs are stopped. Furthermore, the virus has a high mutation rate, which could be a problem for future HIV treatments. Thus, there is need to find new and additional methods to treat HIV. The sites of action for potential new drugs include binding of the virion, entry of the virion, uncoating of virion, translocation of viral complementary DNA into the nucleus, integration of viral cDNA into host cell DNA, inhibition of the gene coding the HIV core protein, and inhibition of budding. Agents that inhibit viral entry are particularly sought after because they can prevent infection.

Papuamides are representatives of a class of marine derived cyclic depsipeptides, including callipeltin A, celebesides A and B, homophymine A, mirabamides, microspinosamide, neamphamide A and theopapuamides. They are all reported to have cytoprotective activity against HIV-1 *in vitro* (Table 2).

Andjelic *et al.* [23] recently determined that anti-HIV action of papuamide A occurs through inhibition of virus entry, which is a three-stage process. At first, the virus attaches to the HIV gp120 recognition particle and binds to the host cell receptor CD4 [56,57], which initiates conformational changes within gp120 to expose a new epitope. This epitope interacts with the cellular chemokine co-receptors CCR5 or CXCR4 [58]. Thereafter, gp120 and gp41 undergo further conformational changes to expose the extracellular region of gp41 [59], in which the *N*-terminal region contains a fusion peptide that inserts itself into the cell membrane [60]. Finally, two helical regions of gp41 fold upon each other, bringing the viral and cellular membranes into close proximity. This structural rearrangement causes lipid mixing and fusion of the two membranes, so that the viral core can enter the host cell [61–73].

For papuamide A, the inhibition of virus entry is not specific to R5 or X4 tropic virus [23]. Papuamide A’s ability to inhibit both the X4 and R5 tropic virus was similar to the mirabamides, which were shown to inhibit fusion [24]. Furthermore, papuamide A inhibition does not target the key proteins involved in the viral entry process such as binding to CD4 or HIV gp120. Instead papuamide A works through direct interaction with the virus, and its virucidal activity appears to be independent of the type of envelope glycoprotein expressed, because papuamide A can inhibit HIV pseudo-type viruses expressing envelope glycoproteins from vesicular stomatitis virus or amphotropic murine leukemia virus. This indicates that the mechanism of viral entry inhibition is not HIV-1 envelope glycoprotein specific. Time delayed addition studies with pseudo-typed viruses have shown that papuamide A inhibits viral infection only at the initial stage of the viral life cycle. Additionally, pretreatment studies revealed that the virus, and not the cell, is the target of papuamide A’s action [23]. At similar concentrations to papuamide A, it was demonstrated that papuamides B also inhibits viral entry. Phosphatidylserine, which is a phospholipid present on the viral membrane, has been proposed to be the target of papuamide B.

A proposed model for the mechanism of virucidal activity for these compounds is based on the membrane targeting mechanism proposed for the sterol-dependent antifungal antibiotic bacillomycin [64]. In this model, the lipopeptide has an aliphatic tail, which inserts itself into the fungal membrane. The interaction is then stabilized through tyrosine binding to sterol present in the fungal membrane. Cholesterol is a major component of the HIV viral membrane due to budding from cellular membrane micro-domains rich in cholesterol and sphingolipids [65,66].

To determine the effects of mirabamides on HIV-1 infection, these compounds were tested against two viral strains in HIV-1 neutralization assays. In addition, mirabamides A-D were tested in an HIV-1 envelope-mediated cell fusion assay to determine whether they act during early steps of infection, namely viral entry (Table 2). Mirabamides A, C, and D potently inhibited HXB2 infection of TZM-bl host cells, while mirabamide B was significantly less active. Relative to HXB2, mirabamides A, C and D showed slightly reduced potency toward SF162, and as with HXB2, mirabamide B only weakly inhibited SF162. Mirabamide A, C and D inhibited HIV-1 in neutralization and fusion assays indicating that these cyclodepsipeptides also can act during early stages of HIV-1 entry [24].

In comparison to papuamide A, mirabamides A-D inhibited HIV-1 envelope-mediated fusion with activities comparable to those observed in neutralization assays. Mirabamide A and papuamide A were the most potent inhibitors in the fusion assay (Table 2), while mirabamides C and D inhibited fusion at low micromolar concentrations. Similar to papuamides A and B, mirabamides also exhibited potent anti-HIV activity toward multiple strains of HIV. Furthermore, the results demonstrated that this class of cyclodepsipeptides acts during HIV-1 membrane fusion, presumably through interaction with HIV-1 envelope glycoproteins [24].

Although recent research has been conducted on the synthesis, biological activity and mode of action of cyclodepsipeptides from marine sponges, the molecular targets that mediate the effects of these natural compounds on viral entry have yet to be identified.

### 2.3. Structure-activity relationship

Common attributes of papuamide A and other anti-HIV active depsipeptides include a tyrosine that can interact with cholesterol and a hydrophobic tail that can insert into the viral membrane, resulting in a virucidal effect. Cooperative binding to phosphatidylserine might preferentially target papuamide A to the viral membrane over the cellular membrane [23]. Similar to papuamide A, celebeside A and B also can inhibit HIV-1 entry (Table 2), while celebeside C, which lacks the phosphoserine residue, is inactive. These data further support the assumption that anti-HIV activity correlates with the presence of phosphoserine [50]. However, while papuamide A selectively binds to phosphatidylserine, binding to phosphatidylserine alone is not sufficient to block viral entry [23].

Papuamide C and D inhibit HIV entry as well, although less potently than A and B. This suggests that the free amino group of the 2,3-diaminobutanoic acid residue of papuamides A and B is not required, although it may contribute to virucidal activity of these compounds [23]. Similarly, mirabamide B is less potent than mirabamides A, C, and D in both neutralization and fusion assays. Mirabamide B is the only of the four cyclodepsipeptides to contain a 2,3-dehydro-2-aminobutanoic acid residue in place of the 2,3-diaminobutanoic acid residue found in mirabamide A, C and D, as well as papuamides A and B. Thus, this residue seems to be important for the anti-HIV activity in this class of cyclodepsipeptides [24].

Mirabamide A, which contains the rhamnosylated β-methyltyrosine residue, was observed to be the most potent of the four mirabamides. Ratnayake *et al.* [55] suggested that the β-methyltyrosine residue is critical for the anti-HIV activity of theopapuamide. However, data obtained with mirabamides suggest that the β-methyltyrosine residue alone is not essential for anti-HIV activity [24]. Rather, these results may indicate that β-methyltyrosine residues bearing a substitution at the 4′ position can be tolerated with no deleterious effects on antiviral activity and the presence of a free 4′ hydroxyl on the tyrosine unit is not essential. One hypothesis that is consistent with results reported for mirabamide A and papuamide A, which both contain a β-methyltyrosine, is that this residue imparts a specific conformation required for binding to target protein/s involved in HIV-1 entry [24]. Theopapuamide, which lacks a β-methyltyrosine residue, was reported to be inactive [24], but later studies demonstrated that theopapuamide B is active in the neutralization assay (Table 2) [50]. Further evidence is given by results obtained with homophymine A (Table 2). The antiviral activity found in homophymine A, in which the β-methoxytyrosine is replaced by an *O*-methyl threonine, ruled out the hypothesis that β-methoxytyrosine is essential for antiviral activity [51].

## 3. Compounds with Anti-tumor Activity

Malignant cancer cells utilize their intrinsic migratory ability to invade adjacent tissues and the vasculature, and ultimately to metastasize. The spread of cancer cells to distant sites in the body is the major cause of death in cancer patient. Therefore, a big challenge in cancer therapy is to inhibit the spread of tumor cells from primary tumor sites to other organs. Various compounds exert anti-migratory effects, and some of them have also been shown to prevent tumor metastasis. Most of their migration-inhibiting effects are based either on interference with tumor adhesion to extracellular matrix components or on the reduction of tumor cell-associated protease activity.

Cell migration occurs through membrane protrusion, adhesion to the extracellular matrix, cell body translocation, and tail retraction. All of these processes imply the role of actin, a key cytoskeletal element that is controlled by proteins both inside and outside of the cell. Disruption of cytoskeleton elements such as microtubules and microfilaments has been shown to interfere with the invasiveness and adhesion of tumor cells during the initial phases of metastasis formation. The actin cytoskeleton constitutes a potential target of interest for anti-cancer drug development [67].

### 3.1. Structural features

**Figure 2:**
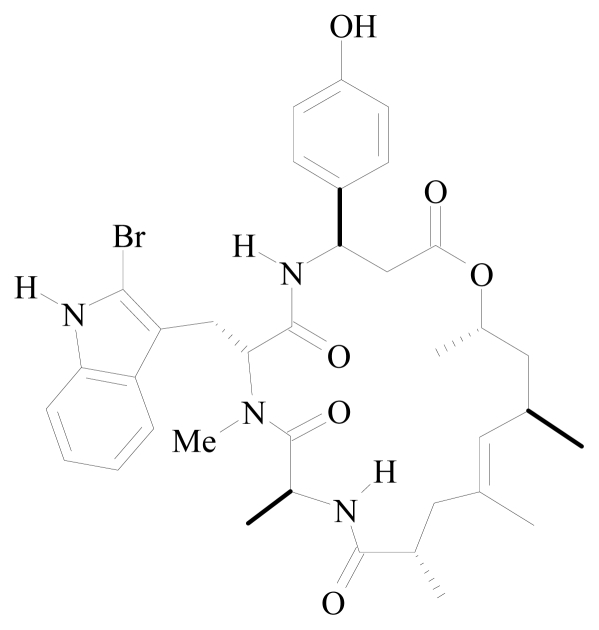
Structure of jasplakinolide.

Jasplakinolide, also known as jaspamide, is a cyclic peptide with a 15-carbon macrocyclic ring containing three amino acid residues, l-alanine, *N*-methyl-2-bromotryptophan, and β-tyrosine (Figure 2). From a structural point of view, jasplakinolide is a representative member of the cyclodepsipeptide family with an l-Ala- d-*N*-Me-2-BrTrp-l-β-Tyr tripeptide fragment, which is linked to a ω-hydroxyacid of polyketide origin, containing four methyl groups, located at 1–3 distance, as well as a trisubstituted double bond [68]. The importance of the d-BrTrp-l-β-Tyr segment in the backbone and the aromatic electrons of the β-tyrosine residue during binding of jasplakinolide to other positively charged species such as metal ions and positively charged amino acid residues of proteins has been demonstrated. Such binding could cause conformational changes in both jasplakinolide and target proteins and affect their properties [69].

Callipeltin A is a macrocyclic lactone containing four amino acids in the L configuration, alanine, leucine, threonine (two residues); one (arginine) in the D configuration; two *N*-methyl amino acids, *N*-methyl alanine and *N*-methyl glutamine; a methoxy tyrosine, a 3,4-dimethyl-l-glutamine; and a 4-amino-7-guanidino-2,3-dihydroxypentanoic acid (AGDHE), formally derived from l-arginine [70].

In addition to their antiviral activity, homophymines [71] and theopapuamide [55] exhibit cytotoxicity against various tumor cell lines. All of these cyclic depsipeptides contain β-methoxytyrosine. Therefore, the desmethoxy analog of callipeltin B was synthesized to evaluate the role of its β-methoxytyrosine residue. It was speculated that cytotoxicity results from the elimination of methanol from β-methoxytyrosine to form a reactive quinone methide intermediate. However, in an MTT assay with HeLa cells, no difference in cytotoxic activity was found between callipeltin B and its desmethoxy analog, suggesting that this intermediate is unlikely to be the principal source of cytotoxicity [47,71].

Spongidepsin has been isolated from the marine sponge *Spongia* sp. Its structure contains 9-hydroxy-2,4,7-trimethyltetradeca-14-ynoic acid and *N*-methylphenylalanine residues joined in a 13-membered ring. Spongidepsin shows cytotoxic activity against J774.A1, WEHI-164 and HEK-293 cancer cell lines with an IC_50_ in the sub-micromolar range [72]. Assignment of absolute stereochemistry and total synthesis were reported [73–75].

Nine new cyclodepsipeptides, homophymines, B–E and A1–E1, were isolated from the sponge *Homophymia* sp. The new structures, featuring new polyketide-derived end groups, display very potent cytotoxic activity with IC_50_-values in the nM range against a panel of human cancer cell lines [76]. A comparison of their activity against different tumor cell lines showed moderate selectivity toward human prostate (PC3) and ovarian (OV3) carcinoma. When their resistant counterpart cell lines were compared (MCF7/MCF7R, HCT116/HCT15, HL60/HL60R), no significant differences were observed, indicating that overexpression of P-gp (MDR1, ABCB1) did not affect the intracellular accumulation of homophymines suggesting that they are not substrates for the efflux pump. In terms of the structure-activity relationship, homophymines A1–E1, featuring the 4-amino-6-carbamoyl-2,3-dihydroxyhexanoic acid residue, exert stronger potency, when compared to the corresponding A–E compounds in which the same residue is in its carboxy form. However, the lack of an effect of homophymines on caspases 3 and 7 activation indicates that homophymines display a toxic rather than antiproliferative activity [76].

### 3.2. Mode of action

In contrast to microtubules, which have been targeted successfully with anti-tumor drugs such as taxol-like compounds and the vinca alkaloids, very few actin-targeting drugs have been characterized. To date, no actin targeting drugs have been used in clinical trials due to their severe cytotoxicity. One reason for this cytotoxicity is that drugs such as cytochalasins and latrunculins disrupt actin microfilaments in both non-tumor and tumor cells [67,77]. However, geodiamolide H and jasplakinolide might be promising candidates. Agents that inhibit Rho GTPases, such as Rac1, may likely be less toxic. Rac1, Cdc42, and Rho A are well known for their role in the regulation of the actin cytoskeleton [78,79].

#### 3.2.1. Actin polymerization

Jasplakinolide, which is a representative member of the cyclodepsipeptide family, is a potent inducer of actin polymerization *in vitro* [80]. Actin is a ubiquitous eukaryotic cytoskeletal protein, critical for many aspects of cell activity. In addition to maintaining cell morphology, it is required for cell motility, cell division, and intracellular transport [81]. Cellular actin rapidly alternates between two forms, monomeric G-actin (globular) and polymeric F-actin (fibrous). The dynamics of the G-actin to F-actin transition may be critical to many cellular functions including cell division. Interestingly, jasplakinolide has a much greater effect on Mg^2+^-actin than on Ca^2+^-actin. Competitive binding studies suggest that jasplakinolide binds to F-actin competitively with phalloidin with a dissociation constant of approximately 15 nM. It was suggested that jasplakinolide might exert its cytotoxic effect *in vivo* by inducing actin polymerization and/or inhibiting depolymerization of actin filaments [80].

The effect of jasplakinolide on the *in vitro* proliferation and differentiation of leukemic cell lines and blast cells from three acute myeloid leukemia patients was comparable to that of cytosine arabinoside. At micromolar concentrations, jasplakinolide suppressed both primary colony formation in agar and the recovery of clonogenic cells from suspension cultures in a dose-dependent manner in the investigated cell lines as well as in fresh blasts (Table 3). In addition, jasplakinolide caused the up-regulation of CD14 and CD11 and down-regulation of CD34 antigens. These results indicate that jasplakinolide significantly inhibits the self-renewing capacity of leukemic progenitors [82].

Exposure of promyelocytic HL-60 cells and human monocytes to jasplakinolide causes a dramatic reorganization of actin from a typical fibrous network to focal aggregates. HL-60 cells exposed to 50 or 100 nM jasplakinolide exhibit a reduced proliferation rate (Table 3). In addition, 100 nM jasplakinolide induces the maturation of HL-60 cells to a granulocyte monocyte lineage. It upregulates the expression of the differentiation markers CD16 and CD14 B. Jasplakinolide also causes the aggregation of F-actin in HL-60 cells and human monocytes. Cultured human monocytes contract and adopt round shapes after treatment with jasplakinolide, and a dose-dependent increase in both total actin and *de novo* synthesized portions of the soluble actin is observed in HL-60 cells. Jasplakinolide disrupts the actin cytoskeleton of normal and malignant mammalian cells with no significant effects on phagocytic activity [83].

Jasplakinolide paradoxically stabilizes actin filaments *in vitro*, but *in vivo* it can disrupt actin filaments and induce polymerization of monomeric actin into amorphous masses. Jasplakinolide markedly enhances the rate of actin filament nucleation. This increase corresponds to a change in the size of actin oligomers that can nucleate filamentous growth from four to approximately three subunits, which is mechanistically consistent with the presence of the jasplakinolide-binding site at the interface of three actin subunits. Because jasplakinolide simultaneously decreases the amount of sequestered actin and augments nucleation, the enhancement of polymerization by jasplakinolide is amplified in the presence of actin-monomer sequestering proteins such as thymosin β_4_. Overall, the kinetic parameters *in vitro* define the mechanism by which jasplakinolide induces polymerization of monomeric actin *in vivo*. The expected consequences of jasplakinolide are consistent with the experimental observations and include *de novo* nucleation resulting in disordered polymeric actin and insufficient monomeric actin to allow for remodelling of stress fibers [84].

Actin filaments in suspension-cultured *Nicotiana tabacum* Bright Yellow 2 cells are disrupted by jasplakinolide in a concentration- and time-dependent manner. When cells are treated with a moderate concentration (150 nM) of jasplakinolide, cortical actin filaments are disrupted preferentially, and actin aggregates in the perinuclear region. At concentrations higher than 400 nM and exposure times longer than 30 min, actin filaments in the cell disappear completely. The effect of jasplakinolide on the actin cytoskeleton is reversible even at high concentrations. Although actin filaments disappear in certain cytoplasmic areas in jasplakinolide-treated cells, jasplakinolide is generally accepted as an actin-polymerizing and actin-stabilizing drug [85].

Geodiamolides A and B present antifungal activity, while geodiamolide H is active against a number of cancer cell lines [86]. The anti-proliferative effects of geodiamolides A, B, H and I were investigated against sea urchin eggs (*Lytechinus variegatus*), and T47D and MCF7 human breast cancer cells lines (Table 3). The anti-proliferative activity against breast cancer cells occurred through the disorganization of actin filaments, while microtubule organization was not affected. Normal cell lines, however, did not show cytoskeletal alterations after treatment with the peptides, which underscores the biomedical potential of such compounds. Small structural alterations in geodiamolides largely affect the rank order of potency in each cell line, indicating different cellular sensitivities depending on the phenotype of the cell. Differences in peptide potencies are associated with an amino acid substitution (alanine *versus* β-tyrosine) or with the presence of bromide or iodide in the phenol ring of the *N*-methyltyrosine moiety [87].

Geodiamolide H selectively interferes with actin cytoskeleton of tumor cells (Hs578T, T47D, MCF7), while having no effect on normal cells (MCF 10A, primary culture human fibroblasts, BRL3A rat liver epithelial cells) [87,88]. This cyclodepsipeptide significantly decreases migration and invasion of Hs578T cells due to modifications in the actin cytoskeleton. As no effect on metalloproteinases has been found, it is suggested that actin is the major target of geodiamolide H in Hs578T cells, and impacts cell motility [88].

#### 3.2.2. Polyploidization

A previous study demonstrated that jasplakinolide at 50 nM exhibits antiproliferative activity and increases CD4 and CD14 surface expression. The exposed cells became multinuclear, and the size and the number of nuclei of the cells increased in a time-dependent manner. In addition, an increased number of metaphase chromosomes were observed. Analysis of the DNA content of HL-60 cells revealed a significant increase in the percentage of cells with increased DNA content. These findings indicate that the jasplakinolide induces polyploidization [89].

#### 3.2.3. Apoptosis

Jasplakinolide induces cell death *via* apoptosis. Jasplakinolide is thought to induce apoptotic cell death through a caspase-3-like protease-dependent pathway. In addition, it has been demonstrated that various transformed cell lines, such as human leukemia Jurkat T cells, murine T lymphoma EL-4 cells, murine myeloma SP-2/0 cells, murine macrophage-like J774.1 cells and murine fibroblast L cells, are more sensitive to jasplakinolide-induced apoptosis than normal, non-transformed cells such as murine thymocytes and spleen T cells [90].

Jasplakinolide-induced loss of viability by programmed cell death in the HL-60 human promyelocytic leukemia cell line is accompanied by neutral endopeptidase/CD10 expression on the surface of the apoptotic cells. HL-60 cells normally do not express detectable amounts of neutral endopeptidase/CD10 on their surface or in the cytoplasm. This implies an association between apoptosis induction and CD10/neutral endopeptidase expression in myeloid cell lines. Moreover, in the promonocytic U937 and mature monocytic THP-1 cell lines, jasplakinolide induced apoptosis but not CD10 expression. In HL-60 cells, CD10 expression was partially, but not totally, blocked by a broad-spectrum caspase inhibitor. Therefore, these results suggest that programmed cell death induced by jasplakinolide occurs *via* caspase-dependent and -independent pathways [91].

Homophymins display very potent cytotoxic activity with IC_50_-values in the nM range against a panel of human cancer cell lines [76]. However, the lack of an effect of homophymines on caspases 3 and 7 activation indicates that homophymines display a toxic rather than antiproliferative activities.

### 3.3. Jasplakinolide and arenastatin A analogs as mechanistic probes

The mechanism of action of depsipeptide-based actin-targeting compounds is quite unclear in terms of the molecular aspects of their interaction with a biological target. It is difficult to explain the similar effects of these metabolites on actin in the absence of a receptor map and additional structural details. Previous investigations on jasplakinolide have indicated that the two structural elements of the molecule, the tripeptidic portion and the polyketide fragment, play a cooperative role in creating the bioactive shape of the compound [97,98]. In fact, it seems that the tripeptide moiety, in the macrocycle, is forced to adopt a preferential β-turn conformation, indicating that the polyketide fragment plays a decisive role in generating geometric constrains and inducing a selective conformation [68]. Synthesis and biological evaluation of modified jasplakinolide analogs were performed to obtain additional information on the potential pharmacophoric core of the target molecule. Although the biological profile of jasplakinolide is established, many mechanistic details are still missing in terms of molecular target identification. For this reason, the natural metabolite was synthetically modified with a β-amino acid in the cyclopeptide backbone, similar to the parent compound [99]. However, the extreme simplified polyketide moiety, which was necessary to allow more synthetic accessibility of analogs, was detrimental to biological activity [68].

**Figure 3:**
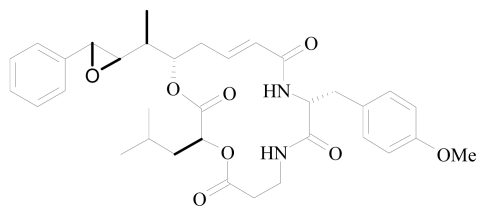
Structure of arenastatin.

Arenastatin A (Figure 3) has an identical structure to cryptophycin-24, which was isolated from the cyanobacterium *Nostoc sp.* [100]. The cyclodepsipeptide was shown to have extremely potent cytotoxic activity (IC_50_ = 5 pg/mL) against KB 3-1 cells. Cytotoxicity is caused by inhibition of microtubule assembly through binding to the rhizoxin/maytansine site on tubulin [93,94]. However, arestatin A was found to exhibit only marginal *in vivo* anti-tumor activity after intravenous administration due to rapid metabolism of the 15,20-ester linkage in arenastatin A. To overcome this biological instability, a 15,20-triamide analog was synthesized, in which the labile ester function is replaced by an amide moiety. This analog was found to show sufficient stability in serum and moderate levels of cytotoxicity (IC_50_ = 6 ng/mL). However, it was almost insoluble in polar solvents, thus it could not be applied for *in vivo* biological evaluation [101]. Therefore, a series of arenastatin A analogs was synthesized [95,102], and two tertiary amine analogs with polar diethylamine and piperazine moieties on the phenyl ring were found to be the most potent cytotoxic compounds (IC_50_ = 0.18 and 1.5 ng/mL, respectively) with good solubility and stability. The *in vivo* anti-tumor activity of the intraperitoneally applied diethylamine analog was tested in subcutaneously implanted murine sarcoma S180 cells (Table 3). The diethylamine analog inhibited the growth of the tumor at a dose of 1 mg/kg with comparable efficacy to that of 5 mg/kg doxorubicin, without signs of acute toxicity [95].

## 4. Cyclodepsipeptides as Pharmacological Tools

### 4.1. Tools for studying actin organization and dynamics

Jasplakinolide is a potentially useful pharmacological tool for the study of actin organization and dynamics in living cells, since it induces actin polymerization *in vitro* and, unlike phalloidin, is membrane permeable. Jasplakinolide continues to be employed in studies investigating the role of the actin cytoskeleton in various cell processes. For example, jasplakinolide induces the formation of the acrosomal process in a protozoan parasite [103]. Stabilization of actin filaments by jasplakinolide arrests oocyte maturation, cortical granule exocytosis, sperm incorporation, cone resorption, and cell cycle progression during fertilization in mice [104]. When treated with jasplakinolide, morphological changes in the Golgi complex are blocked, suggesting that they are correlated with actin cytoskeleton rearrangements [105]. Jasplakinolide’s inhibition of actin filament disassembly was employed to reveal the role of actin depolymerization in lamellipodial protrusion [106].

Participation of the actin cytoskeleton in the transduction of proliferative signals has been established through the use of compounds that disrupt the cytoskeleton. To address the possibility that actin also participates in the transduction of apoptotic signals, the response of the murine interleukin 2 (IL-2)-dependent T cell line CTLL-20 to treatment with jasplakinolide upon IL-2 deprivation was studied. Treatment of CTLL-20 cells with jasplakinolide, in the presence or absence of recombinant human IL-2, altered actin morphology. Jasplakinolide was not toxic to CTLL-20 cells, nor was apoptosis induced in the presence of exogenous recombinant human IL-2. However, actin stabilization at the time of IL-2 deprivation enhanced apoptosis by changing the kinetics of apoptotic commitment. This effect of jasplakinolide correlated with its ability to stabilize polymerized actin, as treatment with a synthetic analog of jasplakinolide with a greatly reduced ability to bind actin, jasplakinolide B, did not enhance apoptosis. This enhancement occurred upstream of the induction of caspase-3-like activity and could be inhibited by the overexpression of the anti-apoptotic protein Bcl-x_L_. These data suggest that the actin cytoskeleton plays an active role in modulating lymphocyte apoptosis induced by cytokine deprivation [107].

Jasplakinolide markedly influences the morphogenetic process in the green alga *Micrasterias* when used at concentrations higher than 3 μM. Upon treatment, the development of *Micrasterias* is inhibited or strongly retarded, malformations occur, and large vacuoles are formed. At the ultrastructural level, dense, abnormal accumulations of filamentous structures have been found, indicating actin filament polymerization *in situ*. Moreover, the displacement of organelles and aggregates of endoplasmic reticulum cisternae were observed, but microtubule arrangement and microtubule-dependent processes remained undisturbed. Cells allowed to recover from jasplakinolide treatment continued their growth, but showed severe changes in the cell pattern and displacement of organelles, suggesting that even after removal of the drug, some basic features for the morphogenetic process remained altered. Taken together, jasplakinolide is a powerful tool for investigations into actin-dependent processes [108].

Whereas the actin cytoskeleton is a key component involved in cell migration, agents targeting actin dynamics have been investigated. Consequently, valuable *in vitro* pharmacological tools are needed to selectively identify agents that target actin dynamics. In response to the absence of any standardized process, Hayot *et al.* [67] developed a multi-assay strategy for screening actin-affecting drugs with anti-migratory potentials. For validation, they used the cancer cell lines MCF7 and A549, and the three actin-affecting drugs cytochalasin D, latrunculin A, and jasplakinolide. The effects of these drugs on the kinetics of actin polymerization were quantified in tubes by spectrofluorimetry, and on the dynamics of actin within whole cells by fluorescence microscopy. Using quantitative videomicroscopy, they investigated the effects of the drugs on cell motility. Finally, the combined drug effects on cell motility and cell growth were evaluated by means of a scratch-wound assay. While the results showed concordant drug-induced effects on actin polymerization *in vitro* in test tubes and within whole cells, the whole cell assay was more sensitive than the tube assay. The inhibition of actin polymerization induced by cytochalasin D was paralleled by a decrease in cell motility for both cell types. However, jasplakinolide significantly enhanced the locomotion of A549 cells, while it significantly inhibited the movement of MCF-7 cells. All these effects were confirmed by the scratch-wound assay, except for the effects of jasplakinolide on MCF-7 cell motility. These events were later compensated by additional effect that occurred during wound recolonization, possibly through effects on the cell growth features. Therefore, the use of multi-assays with different levels of sophistication and biological relevance is recommended for the screening of new actin-affecting drugs with potentially anti-migratory effects [67].

### 4.2. Tools for studying the role of inhibition of Na^+^/Ca^2+^ exchange

In cardiac sarcolemmal vesicles, callipeltin A induces a powerful (IC_50_ = 0.85 μM) and selective inhibition of the Na^+^/Ca^2+^ exchanger. In electrically driven guinea-pig atria, callipeltin A causes a positive inotropic effect, which is accompanied by a rise in resting tension at high concentrations. Thus, the positive inotropic effect is linked to the inhibition of the Na^+^/Ca^2+^ exchanger, making callipeltin A a useful tool for studying the role of the cardiac Na^+^/Ca^2+^ exchanger in physiological and pathological conditions [70]. Furthermore, it was reported that the effect of callipeltin A on cardiac and vascular preparations is linked to sodium ionophoric action [109].

### 4.3. Tools for studying cAMP-dependent transepithelial Cl^−^ secretion

Previous studies have shown that cAMP-dependent transepithelial Cl^−^ secretion in the intestinal cell line T84 is reduced upon treatment with phalloidin, an effect that is partly attributed to inhibition of basolateral Na^+^-K^+^-2Cl^−^ cotransport. However, secretory responses are preserved in cells treated with the microfilament disrupter cytochalasin D. Jasplakinolide inhibits cAMP-dependent secretion and Na^+^-K^+^-2Cl^−^ cotransport. Latrunculin A, which sequesters G-actin monomers, profoundly alters the distribution of F-actin and reduces basal transepithelial resistance with minimal effect on secretion. Cytochalasin D, but not latrunculin A, activates Na^+^-K^+^-2Cl^−^ cotransport. These observations have provided further evidence that vectorial ion transport is influenced by the cytoskeleton and support a model in which disassembly of F-actin by specific pharmacological agents or in response to secretory agonists favors activation of Na^+^-K^+^-2Cl^−^ cotransport [110].

## 5. Conclusions

Most of the cyclodepsipeptides that are currently evaluated in clinical trials are used in refractory cancer therapy. They include kahalalide F and aplidin (plitidepsin), which were isolated from mollusks or green algae, and tunicate, respectively. They have shown promising results in phase I and II clinical trials, especially when administered in combination with other chemotherapeutic agents. At present, the biological activity of cyclodepsipeptides from marine sponges remains to be validated in animal studies, and clinical trials are lacking. In cell-based assays, interesting mechanisms of the compounds have been found such as the inhibition of HIV-virus entry (e.g., mirabamides and papuamide A) and actin polymerization (in particular jasplakinolide). Progress has also been made in structure-activity relationship studies, but only small quantities of these natural compounds can be isolated and purified, and this has made the *in vivo* validation of these compounds difficult. For instance, didemnin B, a cyclodepsipeptide isolated from a tunicate, is an example of an *in vitro* effective compound that failed to show anticancer activity at non-toxic concentrations in humans. Therefore, structural determination, structure-activity relationships and the elaboration of large-scale total synthesis of marine sponge derived compounds are required for pharmacodynamic, pharmacokinetic and toxicological evaluation of marine sponge-derived cyclodepsipetides. However, despite the lack of therapeutic validation of these compounds, they remain valuable molecular probes for investigating new signaling pathways.

## Figures and Tables

**Table 1:** Structural characteristics of sponge-derived cyclodepsipeptides.

Cyclo-Depsipeptide	Marine Sponge	Uncommon Residues		
		Amino acids	Polyketide moieties	Ref.
Callipeltin A	Callipelta sp.	AGDHA 3,4-DiMeGln β-OMeTyr		[40] [43] [45] [49]
Celebesides	Siliquariaspongia mirabilis	ACPA 3,4-DiMeGln 3-CThr pSer (Cel. A, B)	DDTD HTMOA	[50]
Homophymine A	Homophymia sp	ADHA AHDMHA 3,4-DiMeGln ThrOMe	HTMOA	[51]
Microspinosamide	Sidonops microspinosa	*N*-MeGln HBPA		[52]
Mirabamides	Siliquariaspongia mirabilis	Dab (except M. B) 3,4-DiMeGln β-OMeTyrRP ClHPr	DHTDA	[24]
Neamphamide A	Neamphius huxleyi	AGDHA 3,4-DiMeGln *N*-MeGln β-OMeTyr	HTMHA	[44]
Papuamides	Theonella mirabilis and Theonella swinhoei	Dab 3,4-DiMeGln hPro β-OMeTyr 3-OHLeu	DHTDA	[48] [53] [54]
Theopapuamides	Geodia barretti and Siliquariaspongia mirabilis	ACPA AMDHA (Th. B) AMTHA (Th. A) 3,4-DiMeGln *N*-MeGln hiLeu (Th. C)	HTMOA	[55]

Abbreviations:
ACPA 3-acetamido-2-aminopropanoic acid ADHA 4-amino-2,3-dihydroxy-1,7-heptanoic acid AGDHA 4-amino-7-guanidino-2,3-dihydroxyheptanoic acid AHDMHA 2-amino-3-hydroxy-4,5-dimethylhexanoic acid AMDHA 4-amino-2,3-dihydroxy-5-methylhexanoic acid AMTHA 4-amino-2,3,5-trihydroxy-5-methylhexanoic acid ClHPr 4-chlorohomoproline 3-CThr 3-carbamoyl threonine Dab 2,3-diaminobutanoic acid DDTD 7,9-dihydroxy-8,10-dimethyltrideca-2,4-dienoic acid DHTDA 2,3-dihydroxy-2,6,8-trimethyldeca-4,6-dienoic acid 3,4-DiMeGln 3,4-dimethyl-L-glutamine hiLeu homoisoleucine hPro homoproline HBPA β-hydroxy-p-bromophenylalanine HTMOA 3-hydroxy-2,4,6-trimethyloctanoic acid HTMHA 3-hydroxy-2,4,6-trimethylheptanoic acid *N*-MeGln *N*-Methylglutamin β-OMeTyr β-methoxytyrosine β-OMeTyrRP β-methoxytyrosine 4′-*O*-α-L-rhamnopyranoside 3-OHLeu 3-hydroxyleucine pSer phosphoserine ThrOMe *O*-methyltreonine

**Table 2:** Anti-HIV activity of cyclodepsipeptides.

Cyclo-Depsipeptide	Assay	Anti-HIV Activity (IC_50_)	Cytotoxicity (TC_50_)	Ref.
Callipeltin A	MTT cell viability on CEM4 lymphocytic cell lines infected with HIV-1 (Lai strain, X4 tropic)	0.01 μg/mL	0.29 μg/mL	[49]

Celebesides	single-round HIV-1 infectivity assay against viruses pseudo-typed with HIV-1 SF162 Envelope	A: 1.9 μg/mL B: >50 μg/mL		[50]

Homophymine A	production of HIV-1 virus (III B strain) measured by quantification of reverse transcriptase activity associated with the virus particles in PBMC cell lines	75 nM	1.19 μM	[51]

Microspinosamide	XTT-based cell viability assay in HIV-1 infected CEM-SS target cells	0.2 μg/mL	3.0 μg/mL	[52]

Mirabamides A-D	HIV-1 neutralization assays:	HXB2:		[24]
	HXB2 (T-cell-tropic) and SF162 (macrophage-tropic) viral strains;	A: 140 nM		
	TZM-bl host cells (expressing CXCR4, CCR5, and CD4)	B: >50 μM		
		C: 140 nM		
		D: 190 nM		
		SF162:		
		A: 400 nM		
		C, D: 1 μM		
	HIV-1 envelope-mediated cell fusion assay	A: 41 nM		
		C, D: low μM range		

Neamphamide A	XTT-based cell viability assay: human T-cell line CEM-SS infected with HIV-1_RF_	28 nM	260 nM	[44]

Papuamide A	HIV-1 envelope-mediated cell fusion assay	73 nM		[24]
	MTT cell viability test	71 nM		[23]
	Virion based fusion assay: co-transfection of pMM310 (plasmid encoding β-lactamase), pAdV Antage (vector), pNL4-3 (X4 tropic proviral DNA) or pNL(AD8) (R5 tropic proviral DNA) into 293FT cells; HeLaT4 or TZM-bl host cells	114 nM		[23]
	Pseudo-type virus assay: pDHIV-3 plus envelope glycoprotein plasmids transfected to 293FT cells; Target cells: CEM-SS or CEM.NKR-CCR5 cells	178 nM		[23]
	tetrazolium-based assay CEM-SS T-cells	3.6 ng/mL	74 ng/mL	[53]

Theopapuamide B	single-round HIV-1 infectivity assay against viruses pseudo-typed with HIV-1 SF162 Envelope	0.8 μg/mL		[50]

Abbreviations:
IC_50_ half-maximal concentration for cytoprotective activity against HIV-infection TC_50_ concentration for 50% reduction in cell viability (half-maximal concentration for cytotoxic response)

IC_50_ and TC_50_ values were not calculated by use of the same assay and exposure time.

“A”, “B”, “C” and “D” in the column “anti HIV activity” correspond to celebesides A and B, and mirabamides A, B, C and D in the respective rows.

**Table 3:** Anti-tumor activity of sponge-derived cyclodepsipeptides.

Cyclodepsipeptide	Marine sponge	Cell line	Growth inhibitory concentration (IC_50_)	Ref.
Arenastatin A (cryptophycin –24)	*Dysidea arenaria*	KB 3-1	5 pg/mL	[92] [93] [94]
diethylamine analog		S180	0.18 ng/mL	[95]

Geodiamolides	*Geodia sp.* and *Geodia corticostylifera*			[86]
		sea urchin eggs;	≈100–600 nM	[87]
		T47D and MCF7	≈20–115 nM	[87]
		Hs578T	120 nM	[88]

Homophymines	*Homophymia sp.*	PC3, OV3, MCF7/MCF7R, HCT116/HCT15, HL60/HL60R	2–100 nM	[76]

Jasplakinolide (jaspamide)	*Jaspis sp.* and *Hemiastrella minor*	HL-60	100 nM	[83]
			50 nM	[89]
			100 nM	[91]
			100 nM	[96]
		Jurkat T cells, EL-4, SP-2/0, J774.1	2 μg/mL	[90]

Spongidepsin	*Spongia sp.*	J774.A1	0.56 μM	[72]
		WEHI-164	0.42 μM	
		HEK-293	0.66 μM	

Theopapuamides	*Geodia barretti* and *Siliquariaspongia mirabilis*	CEM-TART HCT-116	0.5 μM	[55]
			0.9 μM	
		HCT-116	Theop. A: 2.1 μg/mL	[50]
			Theop. B: 4.0 μg/mL	
			Theop. C: 2.1 μg/mL	
